# Luteal phase ovarian stimulation following oocyte retrieval: is it helpful for poor responders?

**DOI:** 10.1186/s12958-015-0076-2

**Published:** 2015-07-25

**Authors:** John Zhang

**Affiliations:** Reproductive Endocrinology and Infertility, New Hope Fertility Center, 4 Columbus Circle, New York, NY 10019 USA

**Keywords:** Luteal phase stimulation, Double ovarian stimulation, Poor ovarian response, Diminished ovarian reserve, IVF

## Abstract

**Background:**

Poor ovarian response and retrieval of no oocytes following ovarian stimulation for *in vitro* fertilization (IVF) is a challenging problem for both the patient and the clinician.

**Findings:**

Recent evidence indicates that folliculogenesis occurs in a wave-like fashion indicating that there are multiple follicular recruitment waves in the same menstrual cycle. This relatively new scientific concept provides new opportunities for the utilization of ovarian stimulation in women with poor ovarian response. This communication reports on the social and scientific rationale for the use of luteal phase ovarian stimulation following oocyte retrieval in the same cycle (also called double stimulation).

**Conclusions:**

Data to date showed that double ovarian stimulation in poor responders provides shorter time for retrieving mature oocytes with the potential formation of good quality embryos, and thus healthy pregnancies.

## Introduction

Poor ovarian response after ovarian stimulation for *in vitro* fertilization (IVF) is a challenge for the patients and their clinicians. Currently, ovarian stimulation in women with diminished ovarian reserve (DOR) has been individualized due to the development of a variety of ovarian stimulation protocols [1, 2]. Many studies have evaluated the use of various ovarian stimulation regimens to improve the outcome of poor responders undergoing IVF treatment; some of these protocols involve minimal/mild ovarian stimulation [3]. Additionally, patients with poor prognosis have high cancellation rates, along with lower pregnancy and live birth rates. These relatively high cancellation rates are leading clinicians and researchers to question whether IVF with conventional “follicular phase” ovarian stimulation has alternative approaches in the DOR population [4].

Recent evidence indicates that folliculogenesis occurs in a wave-like fashion [5]. Thus, in the same menstrual cycle, there are multiple follicular recruitment waves [5]. This fact challenges the traditional theory that a single cohort of antral follicles only develop during the follicular phase of the menstrual cycle [6]. It also provides other new opportunities for clinicians to utilize ovarian stimulation especially in women with DOR [6]. This manuscript reports on the social and scientific rationale for the use of luteal phase ovarian stimulation following oocyte retrieval, in the same cycle (also called double ovarian stimulation) when follicular phase ovarian stimulation was already undergone. It also addresses special situations where luteal phase stimulation could represent an alternative for patients with DOR when no oocytes were retrieved in the conventional “follicular phase” ovarian stimulation IVF.

## Findings

### Social and Scientific basis for luteal phase ovarian stimulation

It is not uncommon to have no oocytes retrieved in women with DOR undergoing IVF especially when there is very little follicular growth [7]. In these situations, no oocytes are usually retrieved from apparently normally growing ovarian follicles with normal estradiol concentrations despite meticulous repeated follicular aspiration and flushing [8]. Reasons for this include poor ovarian response, errors in human chorionic gonadotrophin (hCG) administration/timing for final oocyte maturation, underlying ovarian dysfunction, or premature ovulation [9–11]. In spite of these reasons, obtaining no oocytes following oocyte retrieval represents a tremendous stress for the patient. Luteal phase stimulation could potentially alleviate some of this stress since the patients do not have to wait for another menstrual cycle before undergoing ovarian stimulation.

Folliculogenesis is an ongoing process in which multiple follicles are in the process of development [12, 13]. The traditional concept of folliculogenesis supports the recruitment of various antral follicles in each ovary from the “late luteal phase” of the preceding menstrual cycle to the following follicular phase [12]. Interestingly, there is increasing evidence to indicate that multiple waves of antral follicles develop during the same woman’s menstrual cycle challenging the concept of single recruitment episode during the follicular phase [12, 14].

Three theories of antral follicle recruitment have been suggested [12]. The first theory of “continuous recruitment” suggests that small antral follicles grow and regress constantly throughout the inter-ovulatory (between two ovulatory events) interval and the dominant ovulatory follicle is selected by chance from the pool following luteal regression [12]. The second theory of a “single recruitment episode” suggests that an ovulatory follicle is selected from a single follicular cohort that emerges following luteal regression [12]. The third “wave theory” of follicle recruitment suggests that two or more cohorts of antral follicles are recruited during the same ovarian cycle. The dominant follicle that develops in the final wave of the inter-ovulatory interval ovulates while preceding waves are anovulatory [12]. The “wave theory” of follicle recruitment is the basis of the luteal phase ovarian stimulation protocol. Whether synchronization of the emergence of follicular waves with ovarian stimulation produces more competent oocytes and healthier embryos and whether it enhances the efficiency of assisted reproductive technology in women with DOR remains to be determined [15].

### Luteal phase ovarian stimulation protocol and review of pertinent studies

**Fig. 1:**
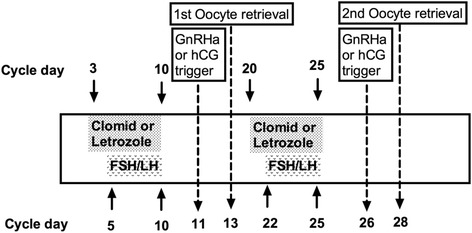
Double ovarian stimulation protocol: both follicular and luteal phase ovarian stimulation in the same cycle. GnRHa, gonadotropin releasing hormone agonist; hCG, human chorionic gonadotropin; FSH, follicle-stimulating hormone; LH, luteinizing hormone

A typical protocol of luteal phase ovarian stimulation starts 2–7 days following oocyte retrieval during the same menstrual cycle. Following oocyte retrieval, a second ovarian stimulation is usually started with low dose gonadotropins (75–150 IU/day) and either clomiphene citrate (25–100 mg/day) or letrozole (2.5–5 mg/day) when the lead follicle measures less than 13 mm [16]. After 5 days of stimulation, ultrasound and blood hormone monitoring is resumed as usual. Human chorionic gonadotropins (5000 IU) or GnRH agonist (0.1 mg) is then used for oocyte maturation when the lead follicle becomes larger than 18 mm (Fig. 1). Because clomiphene citrate could cause a thin endometrial lining [17, 18], and because the endometrium is out of phase following luteal phase stimulation, embryo freezing is usually recommended followed by a frozen embryo transfer in a subsequent cycle [19].

A randomized, controlled study investigated the benefit of luteal phase ovarian stimulation in patients who previously had a suboptimal ovarian stimulation cycle [20]. In that study, 40 patients with poor ovarian response, defined as having 3–6 retrieved oocytes in their previous cycle, were included. Compared to women who had recombinant human gonadotropins in the early follicular phase, women who had luteal phase stimulation had similar number of oocytes retrieved [20]. Although, that study indicated that poor responders do not benefit from commencing recombinant human FSH therapy in the luteal phase, it demonstrated that luteal phase stimulation could produce a reasonable number of oocytes thus representing an alternative to follicular phase stimulation in poor responders. However, that study did not evaluate double ovarian stimulation in the same menstrual cycle and it did not assess clinical or live birth rates.

Another randomized, open-label pilot trial (total *n* = 18) investigated whether luteal phase initiation of gonadotropins would improve oocyte yield compared with follicular phase administration in women (age range 20–42) with poor ovarian response [21]. In that trial, poor ovarian response in a previous cycle was defined as either less than 5 follicles on day of HCG administration, or less than 5 oocytes retrieved, or previous cancelled IVF cycle. Gonadotropins were administered during either the follicular phase (*n* = 9) or the mid-luteal phase of the preceding menstrual cycle (*n* = 9). The number of oocytes retrieved was similar regardless of the stimulation phase. Other endpoints including follicular growth, serum estradiol levels, and pregnancy and live birth rates did not differ between the luteal or follicular phase stimulation [21]. That study indicated that luteal phase initiation of gonadotropins is a safe and potential alternative protocol in poor responders when other protocols have failed.

A recent pilot study evaluated the efficacy of double ovarian stimulation during the follicular and luteal phases in women (*n* = 38, mean age = 36) with poor ovarian response undergoing mild ovarian stimulation IVF [16]. After the first oocyte retrieval, gonadotropins and letrozole were co-administrated to stimulate follicular development. Oocyte retrieval was carried out a second time when dominant follicles had matured following a GnRH agonist trigger. The number of oocytes retrieved was 1.7 ± 1.0 in the first retrieval (follicular phase ovarian stimulation) versus 3.5 ± 3.2 in the second retrieval (luteal phase ovarian stimulation) (*p* = 0.001). Additionally, the number of mature (metaphase II) oocytes was 1.4 ± 1.0 versus 2.7 ± 2.7 (*p* = 0.008) in the first and second oocyte retrievals respectively [16]. From the double stimulation, 167 oocytes were collected and 26 out of 38 (68.4 %) succeeded in producing 1–6 viable embryos cryopreserved for later transfer. Twenty-one women underwent 23 cryopreserved embryo transfers, resulting in 13 clinical pregnancies. This study underscores the usefulness of luteal phase stimulation in assisted reproductive technology.

A recent case report of a patient (aged 41) who had severe DOR as manifested by antral follicle count of 2 also showed success with double ovarian stimulation [16]. That patient underwent minimal ovarian stimulation IVF protocol (using clomiphene citrate with low dose gonadotropins). One mature oocyte leading to a good embryo was successfully obtained through luteal phase ovarian stimulation following failure to retrieve any oocytes by minimal ovarian stimulation in the conventional follicular phase ovarian stimulation earlier in the same menstrual cycle [16]. This case reinforces the fact that luteal phase stimulation represents a clinically valuable alternative when no oocytes were retrieved following IVF with follicular phase ovarian stimulation.

## Conclusion

Double ovarian stimulation (follicular and luteal phases) in the same menstrual cycle could provide an opportunity to retrieve more oocytes, potentially producing a pregnancy in poor responders in a shorter period of time. Although luteal phase ovarian stimulation does not seem to be better than follicular phase ovarian stimulation, it could produce a positive (good embryo) outcome in women with DOR when no oocytes were retrieved in the same conventional IVF cycle. This, without doubt, alleviates some of the stress that poor responders have following a “no egg” results. Additionally, the resulting embryos from luteal phase ovarian stimulation protocols could have similar developmental potential and could produce clinically acceptable pregnancy rates when compared to embryos produced by follicular phase ovarian stimulation. Double stimulation thus represents a promising approach for patients with poor ovarian response especially when conventional IVF protocols have failed or when time is of the essence. It is unfortunate that studies to date included small sample sizes. Large cohort studies/randomized clinical trials with live-birth rates as outcomes are needed to better elucidate the benefit of luteal phase (and double) ovarian stimulation in the DOR population.
